# Association Between a Two-Cycle Plan-Do-Study-Act Quality Improvement Intervention and Biomedical Waste Segregation Compliance in a Large Indian Government Tertiary Teaching Hospital: A 51-Month Interrupted Time Series Study

**DOI:** 10.7759/cureus.116595

**Published:** 2026-09-20

**Authors:** Vanini Jowel, Shailesh Tripathi, Anusha Chouhan, Kumari Seema

**Affiliations:** 1 Hospital Administration, Rajendra Institute of Medical Sciences, Ranchi, IND; 2 General Medicine, Pt. Jawahar Lal Nehru Memorial Medical College, Raipur, IND; 3 Microbiology, Rajendra Institute of Medical Sciences, Ranchi, IND

**Keywords:** biomedical waste management, hospital administration and healthcare management, interrupted time series, needle-stick and sharps injury, plan-do-study-act, waste segregation

## Abstract

Background: Despite guidelines, Indian public sector hospitals continue to face challenges in biomedical waste (BMW) mismanagement, and various gap assessments at the national level show uneven segregation practices. Needle-stick injuries (NSIs), an occupational hazard for healthcare workers (HCWs) in developing countries, have been found to have an incidence of 8.9-10.4 events per 100 beds per year in Indian tertiary care hospitals. As far as we can judge, no study from a large Indian government tertiary setting has evaluated the incidence of NSIs concurrently with Plan-Do-Study-Act (PDSA) cycles for BMW management using interrupted time series (ITS) analysis.

Objective: This study aimed to evaluate the association between a two-cycle PDSA-driven quality improvement (QI) intervention and (i) BMW segregation compliance and (ii) NSI incidence per 100 occupied bed-years using 51 months of prospective hospital data.

Methods: We conducted a quasi-experimental ITS regression study at a government tertiary teaching hospital (2,327 beds, 95% occupancy, approximately 2,210 occupied beds) in Jharkhand, India. A concurrent control was not feasible because the intervention was implemented hospital-wide as part of preparation for National Accreditation Board for Hospitals and Healthcare Providers (NABH) accreditation. Primary data were drawn from monthly weighbridge logs, periodic waste composition audits, structured audit checklists, and a manual paper-based incident register over 51 consecutive months (January 2022 to March 2026); the intervention began in April 2025. The primary model was segmented ordinary least squares regression with Newey-West standard errors; sensitivity analyses incorporated seasonal harmonic adjustment, lag variation, and alternative autocorrelation specifications.

Results: Segregation compliance showed a significant immediate level increase of 14.3 percentage points (pp) at intervention onset (β₂=14.31; 95% CI: 9.55-19.08; p<0.001) and a positive post-intervention slope change of 2.57 pp per month (β₃=2.567; 95% CI: 1.981-3.152; p<0.001). The pre-intervention trend was flat to slightly negative (β₁=-0.092; p=0.066), and model fit was high (R²=0.939). All four sensitivity analyses were directionally consistent. The descriptive decline in NSI (2.08-0.33 events per month) was not confirmed by the log-linear ITS model (β₂ p=0.397; β₃ p=0.270) and is therefore reported as an associative trend only. Knowledge scores improved across all cadres (p<0.001), with the largest gains among sanitation staff (+37.8 pp) and waste handlers (+36.3 pp).

Conclusions: The PDSA intervention improved BMW segregation compliance, which was significant and sustained over time. Without a simultaneous control group and given institutional confounders, enormous caution in referring to the impacts as causal remains advised. Caution should be exercised in the interpretation of NSI trends as they are not statistically significant and are based on passive reporting. The results indicate that the PDSA-based frameworks are feasible in public hospitals.

## Introduction

Biomedical waste (BMW) generated by healthcare facilities poses risks far greater than those posed by municipal solid waste. Annual reporting by the Central Pollution Control Board (CPCB) has documented a rising national trend in BMW generation, from approximately 559 tonnes per day in 2017 [1] to approximately 619 tonnes per day in 2019 [2]; the number of authorized healthcare facilities has also more than doubled over this period. The Bio-Medical Waste Management Rules, 2016, as subsequently amended, prescribe a four-color segregation scheme at the point of generation and place primary responsibility for compliance on the occupier of the healthcare establishment (HCE) [3].

Demonstrating segregation compliance is also a requirement for accreditation by the National Accreditation Board for Hospitals and Healthcare Providers (NABH) [4]. Despite this regulatory architecture, a nationwide gap assessment of Indian healthcare facilities shows that implementation at the point of generation remains highly variable, particularly in government-funded institutions operating under resource constraints [5]. Published institutional audits from Indian tertiary hospitals report widely divergent baseline segregation performance, although some accredited centers demonstrate near-ceiling baseline levels [6,7]. Structured training programs have produced measurable but heterogeneous improvements [8-10].

The consequences of poor segregation extend beyond regulatory non-compliance. Needle-stick injuries (NSIs) are among the most common occupational hazards linked to unsafe sharps disposal, exposing healthcare workers (HCWs) and waste handlers to blood-borne viruses such as human immunodeficiency virus, hepatitis B virus, and hepatitis C virus. Indian tertiary care surveillance data report incidences of 8.9 events per 100 beds per year in a North Indian teaching hospital and 10.4 events per 100 occupied beds per year in a western Indian tertiary center [11,12].

Sharps injuries among HCWs are known to be substantially under-reported; institutional estimates suggest that half or more of such injuries go unreported, and under-ascertainment is likely greater still in settings reliant on voluntary paper-based reporting [13]. Indian tertiary care series have identified nursing, sanitation, and waste handling personnel among the cadres at highest risk, with a substantial proportion of injuries occurring during device handling, recapping, and downstream sharps disposal [14]. A recent cross-sectional study of nurses in an Indian tertiary care center reported that 40.5% had sustained an NSI [15]. Because sharps that are not placed in puncture-proof containers at the point of generation necessarily persist in the downstream waste stream, segregation practice and occupational sharps exposure are mechanistically linked.

Quality improvement (QI) science offers a systematic alternative to single-episode educational campaigns. The Plan-Do-Study-Act (PDSA) cycle, developed from the work of Shewhart and later Deming, is an iterative, data-driven method suited to multi-stakeholder problems requiring short feedback loops [16]. A systematic review of PDSA applications in healthcare has documented both the breadth of the method and considerable variability in the rigor with which it is applied and reported [17]. PDSA cycles have been associated with improved hand hygiene compliance in neonatal intensive care [18] and with reduced medication error rates in an Indian tertiary hospital neonatal unit [19]. To our knowledge, however, the systematic application of a PDSA framework to BMW segregation, with NSI incidence evaluated as a concurrent outcome, has not previously been reported from a large Indian government tertiary setting.

Most available BMW intervention studies from India rely on short pre-post comparisons. Such designs are informative but cannot exclude secular trends, seasonal variation, or concurrent policy change. Interrupted time series (ITS) analysis addresses this limitation by modelling the trajectory of an outcome before and after an intervention, estimating both the immediate level change and the change in slope against the pre-intervention trend [20,21]. Provided that autocorrelation, seasonality, and model specification are addressed explicitly, ITS is widely regarded as the strongest quasi-experimental design for evaluating health system interventions when randomization is not feasible [22]. We identified very few studies applying ITS methodology to BMW management in India and none that included NSI as a concurrent outcome in a large government tertiary setting.

This study had three objectives: (i) to evaluate the association between a two-cycle PDSA intervention and monthly BMW segregation compliance over 51 consecutive months using segmented ITS regression [20,21], (ii) to assess concurrent changes in NSI incidence as a co-primary safety outcome using the same analytical framework, and (iii) to characterize cadre-specific knowledge improvements following structured training [16,17]. The study adds evidence in a methodologically underexplored area, although the single-site design and the absence of a concurrent control group limit the conclusions that can be drawn.

## Materials and methods

Study design and setting

This was a quasi-experimental study with a 51-month ITS design, conducted at the Rajendra Institute of Medical Sciences, a 2,327-bed government tertiary teaching hospital in Ranchi, Jharkhand, India. The hospital provides comprehensive clinical services, including medicine, surgery, obstetrics and gynecology, pediatrics, orthopedics, and intensive care, with an average occupancy of 95% (approximately 2,210 occupied beds). Data were collected prospectively from January 2022 through March 2026 (51 consecutive months). The PDSA intervention was rolled out from April 2025 (month 40 of the observation period), yielding 39 months of pre-intervention baseline data and 12 months of post-intervention follow-up (three months of PDSA Cycle 1, three months of PDSA Cycle 2, and a six-month sustained phase). The study is reported in accordance with the Strengthening the Reporting of Observational Studies in Epidemiology (STROBE) statement for observational studies [23], the Template for Intervention Description and Replication (TIDieR) checklist for intervention description [24], and the Standards for QUality Improvement Reporting Excellence (SQUIRE) 2.0 framework for QI reporting [25].

A concurrent control group was not used for ethical and practical reasons. The hospital administration mandated the intervention as a QI program in preparation for NABH accreditation, and it could not ethically be withheld from any clinical unit [4]. A stepped-wedge cluster design was considered but rejected because the hospital is a single integrated campus with shared waste management infrastructure and the district health department required all government facilities to implement the program simultaneously. No government tertiary hospital of comparable structure exists within 200 km. The best available alternative 39-month pre-intervention observation period analyzed with a segmented ITS model uses the hospital's own baseline trend as the counterfactual. The complete study design, setting, and methodological parameters are summarized in Table 1.

**Table 1:** Study design, setting, and key methodological parameters BMW: biomedical waste; NSI: needle-stick injury; NABH: National Accreditation Board for Hospitals and Healthcare Providers; PDSA: Plan-Do-Study-Act; OLS: ordinary least squares; HAC: heteroskedasticity- and autocorrelation-consistent; IRR: incidence rate ratio; ITS: interrupted time series; STROBE: Strengthening the Reporting of Observational Studies in Epidemiology; TIDieR: Template for Intervention Description and Replication; SQUIRE: Standards for QUality Improvement Reporting Excellence

Parameter	Details
Study design	Pre-post quasi-experimental with 51-month ITS segmented regression
Setting	Government tertiary teaching hospital in Jharkhand, Eastern India
Total bed capacity	2,327 beds
Average occupancy	95% (approximately 2,210 occupied beds)
Study duration	51 consecutive months (January 2022-March 2026)
Pre-intervention period	39 months (January 2022-March 2025)
Intervention period	6 months (April 2025-September 2025): PDSA Cycles 1 and 2
Sustained phase	6 months (October 2025-March 2026)
Primary outcome 1	BMW segregation compliance score (%) via NABH-aligned structured checklist
Primary outcome 2	NSI rate per 100 occupied bed-years (denominator: 2,210 occupied beds)
BMW categories	Yellow (infectious anatomical), Red (recyclable contaminated), White (sharps/metals), and Blue (non-chlorinated plastics/glassware), per BMW Rules 2016
Data sources	Monthly weighbridge records (total BMW weight), periodic waste composition audits, NABH audit checklists, institutional incident register (manual, paper-based)
Digital reporting system	Absent throughout the study period; all NSI reporting via manual paper-based incident register
Junior medical officer rotation	12-monthly (April cohort); new cohort arrived April 2024, 12 months before intervention onset
Primary analysis	ITS segmented OLS regression with Newey-West HAC standard errors (lag 3)
Secondary analyses	Paired t-test, Wilcoxon signed-rank, Poisson IRR, seasonal sensitivity analyses
Statistical software	Stata 17.0; R 4.3.1 (itsa package)
Ethical approval	Institutional Ethics Committee of the Rajendra Institute of Medical Sciences (RIMS) (approval number: 282); individual consent waived
Reporting frameworks	STROBE, TIDieR, SQUIRE 2.0

Data sources

Primary data were extracted from four institutional sources. First, the sanitation department's monthly weighbridge logs recorded the total BMW weight leaving the facility; these logs did not disaggregate weight by regulated stream. Annual totals were measured directly from these logs. Second, periodic waste composition audits were conducted by the sanitation department in each calendar year, during which segregated waste was weighed by regulated stream (Yellow: infectious anatomical waste; Red: recyclable contaminated waste; White: sharps and metallic items; Blue: glassware and non-chlorinated plastics), as categorized under the Bio-Medical Waste Management Rules, 2016 [3]; the year-specific mean stream shares derived from these audits were applied to the measured annual totals to estimate stream-wise annual weights.

Third, a team of three quality officers, all NABH-aligned internal auditors and trained quality assessors, conducted the unannounced audits through a structured observation. They verified segregation compliance in the departments that were randomly selected using a checklist. At least 12 departments were audited each month, with all major clinical areas covered on a rotational basis and each audit noting at least 15 instances of waste disposal per department. The kappa, which checks for inter-rater agreement, was 0.84 at baseline and 0.81 at 20 months with no change in the three-person audit team. The audit showed no evidence of observer drift.

Fourth, NSIs were captured prospectively using the hospital infection control incident register, which captured the date, place, cadre, type of device, and immediate management. The study didn't use a digital incident reporting system at any point in time. All NSI data thus come from a manual, paper-based register. Data from such registers is known to under-report incidents. Thus, other contributions should be considered with caution.

Outcome measures

Two outcomes were of equal importance. The segregated BMW compliance (%) was calculated from a structured NABH-aligned checklist comprising five domains: (1) use of color-coded bags, (2) fill level below three quarters, (3) legible bin labelling, (4) availability of hub cutters in the clinical area, and (5) complete documentation. For every observation, each domain was scored as compliant or non-compliant. The monthly compliance score was determined as the proportion of compliant from all five domains and all departments assessed; all domains were weighed equally, and no post hoc weighting was applied. The number of NSIs was expressed in terms of events per 100 occupied bed-years using 2,210 occupied beds as the denominator. The secondary outcomes were mean waste generation rate (kg per occupied bed per day) of waste by stream and cadre-specific knowledge, attitude, and practice (KAP) assessment scores.

Intervention description

The intervention used a two-cycle PDSA approach implemented over six months (April to September 2025). Each PDSA component, the problem addressed, the responsible team, and the monitoring method are described in Table 2.

**Table 2:** PDSA operational framework: components, actions, and monitoring BMW Quality Committee composition: Medical Superintendent (chair), Infection Control Officer, Sanitation Superintendent, and two rotating departmental representatives. The committee met fortnightly throughout the intervention and sustained phases. BMW: biomedical waste; NSI: needle-stick injury; NABH: National Accreditation Board for Hospitals and Healthcare Providers; PDSA: Plan-Do-Study-Act; JMO: junior medical officer; HCW: healthcare worker; KAP: knowledge, attitude, and practice; SOP: standard operating procedure; ICU: intensive care unit

Phase	Component/activity	Problem identified/action taken	Responsible team	Monitoring method
PDSA Cycle 1 (April-June 2025)	PLAN: Facility audit	Comprehensive NABH-structured checklist audit across all clinical and non-clinical departments; mapped waste stream-specific gaps and infrastructure deficits	Infection Control Officer, Quality Department	Structured audit forms, photographic evidence
	DO: Training (cadre-stratified)	Six training sessions over three months; 312 HCWs trained across four cadres; two-hour sessions; bilingual (Hindi-English) materials; attendance ≥90% per cadre; refresher after Cycle 2 audit	BMW Quality Committee; Medical Education Unit	Pre/post KAP questionnaires; attendance registers
	DO: Infrastructure	Hub cutters and puncture-proof containers installed at ward level; pictographic posters and color-coded bin labels deployed in all departments	Sanitation Superintendent; Engineering	Weekly infrastructure checklist
	STUDY: Performance review	Monthly review of segregation performance and NSI incidence against predefined target indicators; department-level scorecard introduced	BMW Quality Committee (fortnightly meetings)	Monthly audit score; incident register review
	ACT: SOP formalization	SOP formalized; feedback provided to departmental heads; Cycle 1 gaps identified: waste handlers' knowledge lowest (45.5% KAP), low physician engagement (52.8% KAP), bin damage in ICUs, inconsistent labelling in casualty	Medical Superintendent; Quality Committee	SOP register; departmental compliance scorecards
PDSA Cycle 2 (July-September 2025)	Corrective actions from Cycle 1 gaps	Re-training of underperforming cadres (waste handlers, doctors, casualty staff); replacement of damaged bins; reinforcement of supervisory rounds; second round of infrastructure audit	BMW Quality Committee; Departmental Heads	Targeted re-audit; bin replacement log; supervisor round register
Sustained phase (October 2025-March 2026)	Maintenance and institutionalization	Fortnightly BMW Quality Committee meetings continue; department scorecards reviewed; new JMO cohort (April 2025 rotation) incorporated into BMW induction; no major new components introduced	BMW Quality Committee; fortnightly meetings	Monthly audit; scorecard; JMO induction register

Statistical analysis

Primary Analysis: ITS

The ITS segmented regression constituted the primary analytical framework. Monthly BMW compliance scores were modelled using ordinary least squares (OLS) regression, $\mathrm{Y(t)}=\mathrm{&beta;₀}+\text{&beta;₁ (time)}+\text{&beta;₂ (intervention)}+\text{&beta;₃ (time-after-intervention)}+\mathrm{&epsilon;}$, where Y(t) is the outcome at month t, β₀ is the baseline intercept, β₁ is the pre-intervention trend, β₂ is the immediate level change at intervention onset, β₃ is the post-intervention slope change relative to the pre-intervention trend, and ε is the error term [20]. The intervention variable was coded 0 for months 1-39 and 1 for months 40-51; time-after-intervention was coded 0 before the intervention and incremented sequentially (1, 2, …, 12) thereafter.

Compliance is a continuous monthly percentage, making segmented OLS regression the appropriate primary model. NSI, by contrast, is a low-frequency count outcome with several zero-count months; a log-linear model was therefore applied for NSI, acknowledging that OLS assumptions are less appropriate for rare-count data [21,22].

Model diagnostics included residual plot inspection, the Durbin-Watson (DW) statistic, and the Breusch-Godfrey (BG) test for serial correlation. Where significant autocorrelation was detected (DW <1.5 or BG p<0.05), Newey-West heteroskedasticity- and autocorrelation-consistent (HAC) standard errors were computed (lag 3). The Cumby-Huizinga test was applied as a general autocorrelation check, and Cook's D statistics were used to identify influential observations. All p-values were two-tailed (α=0.05).

Secondary Analyses

Secondary analyses were performed for contextual alignment with prior literature only and were explicitly deprioritized in interpretation. Paired t-tests compared mean compliance between the three-month pre-intervention window (January-March 2025) and the three-month post-intervention window (October-December 2025). Wilcoxon signed-rank tests evaluated non-parametric differences in cadre-specific knowledge scores. A Poisson regression with robust standard errors was used to compute the incidence rate ratio (IRR) for NSI, comparing the six-month sustained phase with the six months immediately preceding intervention onset.

Sensitivity Analyses

Four sensitivity analyses were pre-specified: (1) seasonal harmonic adjustment, adding sine and cosine terms at 12-month periodicity; (2) lag sensitivity, varying the assumed intervention onset by one month in either direction (March 2025 and May 2025) to test whether conclusions change under plausible measurement lag; (3) alternative autocorrelation specification, applying a Prais-Winsten AR(1) correction alongside Newey-West lag 3 and lag 6; and (4) NSI model sensitivity, comparing the log-linear OLS model with Poisson, negative binomial, and zero-inflated Poisson models. Analyses were performed using Stata 17.0 (StataCorp LLC, College Station, Texas, United States) and R 4.3.1 (R Foundation for Statistical Computing, Vienna, Austria; itsa package).

Ethical considerations

The study protocol was approved by the Institutional Ethics Committee of the Rajendra Institute of Medical Sciences (RIMS) (approval number: 282). Because the study used routine administrative and observational QI data without patient identifiers, individual informed consent was waived. All audit data were gathered as part of routine NABH accreditation preparatory processes.

## Results

BMW generation profile

A total of 1,801,779 kg of BMW was generated over the 51-month period (Table 3). The measured annual generation rose from 394,304 kg in 2022 to a peak of 442,515 kg in 2023 and then declined slightly in 2024 (416,670 kg) and 2025 (412,837 kg). The mean generation rate was 0.52 kg per occupied bed per day (standard deviation (SD) 0.08, computed across the 51 monthly generation rates rather than the annual averages shown in Table 3), consistent with the 0.5-2.0 kg per bed per day range described for Indian healthcare facilities [26].

**Table 3:** Annual biomedical waste generation by category, 2022-2026 The total column and the per-bed rates are measured values taken directly from the institutional weighbridge logs, which record the total biomedical waste weight only. †Stream-wise values (Yellow, Red, White, Blue) are estimates, apportioned from the measured annual totals using the year-specific mean stream shares derived from the periodic waste composition audits conducted during each calendar year (pooled shares across the study period: Yellow ≈22%, Red ≈22%, Blue ≈55%, and White <0.5%); they were not separately weighed for each calendar year, and the use of year-specific shares explains the small variation in the Yellow percentage across years. *2026 data represent January-March only (three months); the per-bed rate is not shown for this partial year. Kilograms per occupied bed per day were calculated using 2,210 occupied beds (2,327×95%) and the number of days in each year. Waste stream was classified per the Bio-Medical Waste Management Rules, 2016.

Year	Yellow (kg)†	Red (kg)†	White (kg)†	Blue (kg)†	Total (kg)	Yellow (%)	kg/occupied bed/day
2022	88,324	87,536	1,577	216,867	394,304	22.4	0.489
2023	100,008	97,354	1,770	243,383	442,515	22.6	0.549
2024	92,501	93,333	1,667	229,169	416,670	22.2	0.515
2025	89,586	94,540	1,651	227,060	412,837	21.7	0.512
2026*	30,071	30,341	542	74,499	135,453	22.2	-
Total (51 months)	400,490	403,104	7,207	990,978	1,801,779	22.2	0.526

Annual totals in Table 3 are measured values taken directly from the institutional weighbridge logs, which record the total BMW weight only. Stream-wise annual values were not separately weighed for each calendar year and are therefore reported as estimates, apportioned from the measured annual totals using the year-specific mean stream shares derived from the periodic waste composition audits conducted during the corresponding calendar year; the use of year-specific shares explains the small year-to-year variation in the stream percentages. On this basis, the Blue stream (non-chlorinated plastics and glassware) is estimated to account for the largest share (approximately 55% of the total), followed by Yellow (infectious anatomical, approximately 22%) and Red (recyclable contaminated, approximately 22%), with White (sharps and metals) below 0.5%. Such a composition is typical of tertiary care hospitals with high surgical caseloads and high consumption of disposable plastics.

Month-to-month inspection of the measured series showed a consistent seasonal pattern: volumes peaked during July-September (the monsoon period) and in December and were lowest in February-March, tracking fluctuations in patient census. The annual generation profile by stream, together with the generation rate, is presented in Figure 1.

**Figure 1:**
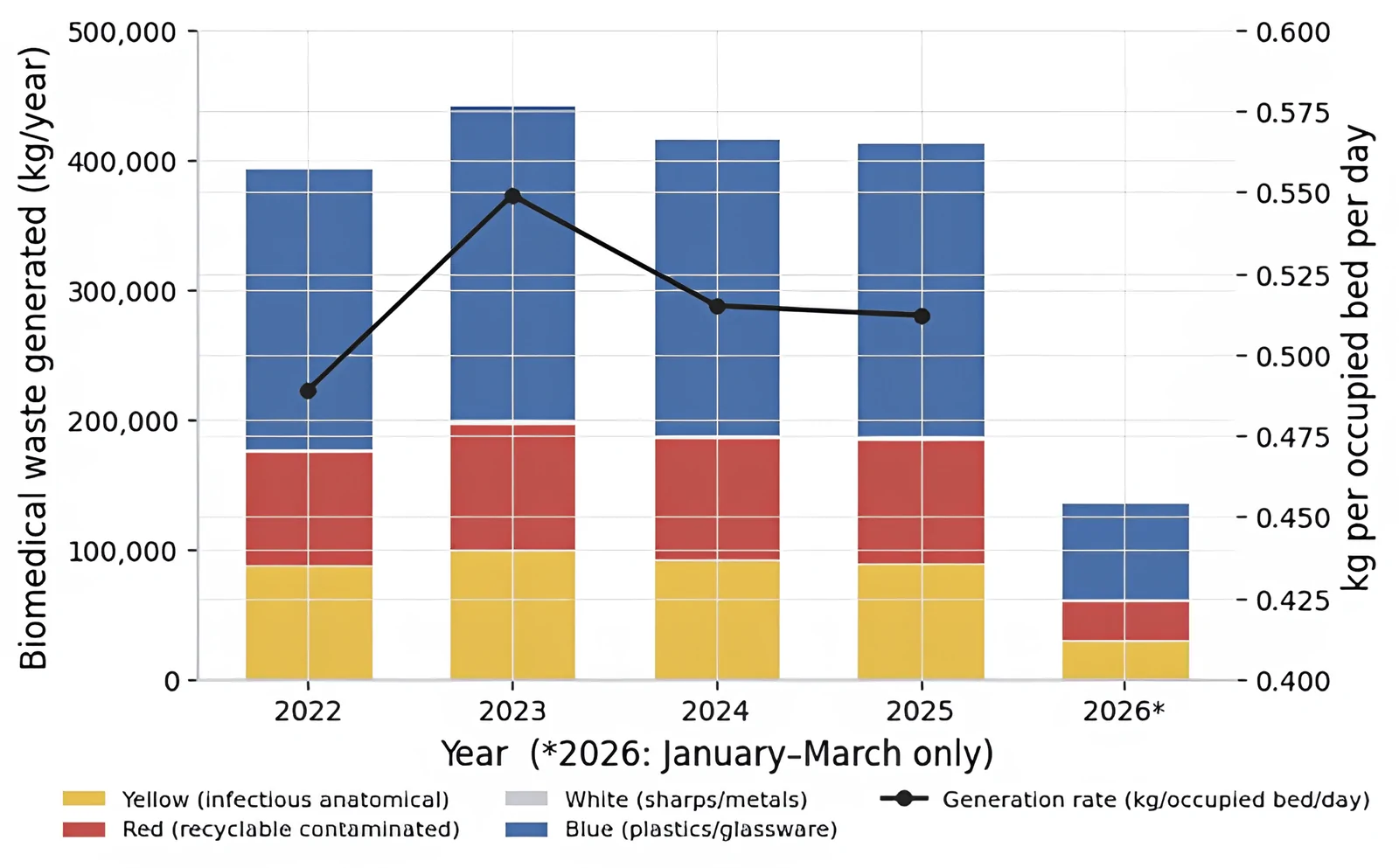
Annual biomedical waste generation by stream and generation rate, 2022-2026 Annual totals are measured from weighbridge logs (total weight only); stream shares are estimates derived from periodic waste composition audits (see Table 3 footnote). 2026 data represent January-March only.

ITS analysis of BMW segregation compliance

The 51-month compliance data segmented regression showed a significant association with the PDSA intervention in the study. Before the intervention, the level of compliance was stable, with a small and non-significant decline (-0.092 pp/month) each month. This pattern suggests that the institution may have drifted in the absence of QI initiatives (Table 4).

**Table 4:** Interrupted time series segmented regression parameters: BMW segregation compliance (primary outcome) *Mild positive autocorrelation detected (DW=1.27); Newey-West HAC standard errors (lag 3) applied, with results unchanged. Model specification: Y(t)=β₀+β₁ (time)+β₂ (intervention)+β₃ (time-after-intervention)+ε. All p-values two-tailed. BMW: biomedical waste; DW: Durbin-Watson statistic; pp: percentage points; HAC: heteroskedasticity- and autocorrelation-consistent

Parameter	Symbol	Coefficient (pp)	SE	95% CI	t-statistic	P-value
Baseline intercept	β₀	58.02	1.12	55.77 to 60.28	51.80	<0.001
Pre-intervention trend (slope)	β₁	-0.092	0.049	-0.190 to 0.006	-1.89	0.066
Level change at intervention onset	β₂	14.31	2.37	9.55 to 19.08	6.04	<0.001
Post-intervention slope change	β₃	2.567	0.291	1.981 to 3.152	8.82	<0.001
Model fit	-	R²=0.939	Adj R²=0.935	F(3,47)=239.33	p<0.001	DW=1.27*

As soon as the intervention began (month 40, April 2025), compliance increased by an estimated 14.31 pp immediately (β₂=14.31; 95% CI: 9.55-19.08; p<0.001), while the slope changed to +2.57 pp per month (β₃=2.567; 95% CI: 1.981-3.152; p<0.001). The model accounted for 93.9% of the variance in monthly compliance (R²=0.939; adjusted R²=0.935; F(3,47)=239.33; p<0.001).

The DW statistic of 1.27 indicates mild positive serial correlation; thus, we use Newey-West standard errors (lag 3). Our estimates remain qualitatively unchanged and quantitatively similar (β₂=14.31; p<0.001; β₃=2.567; p<0.001). The BG test at lag 3 found no substantial residual autocorrelation (χ²=4.82; p=0.186). Moreover, all Cook's D values were below 0.5, suggesting that there are no influential outliers. A model of high R² is a good model. However, it does not mean that institutional confounding variables are not present, nor does it mean that there is a causal effect between protests and government actions. The fitted segmented regression is depicted in Figure 2.

**Figure 2:**
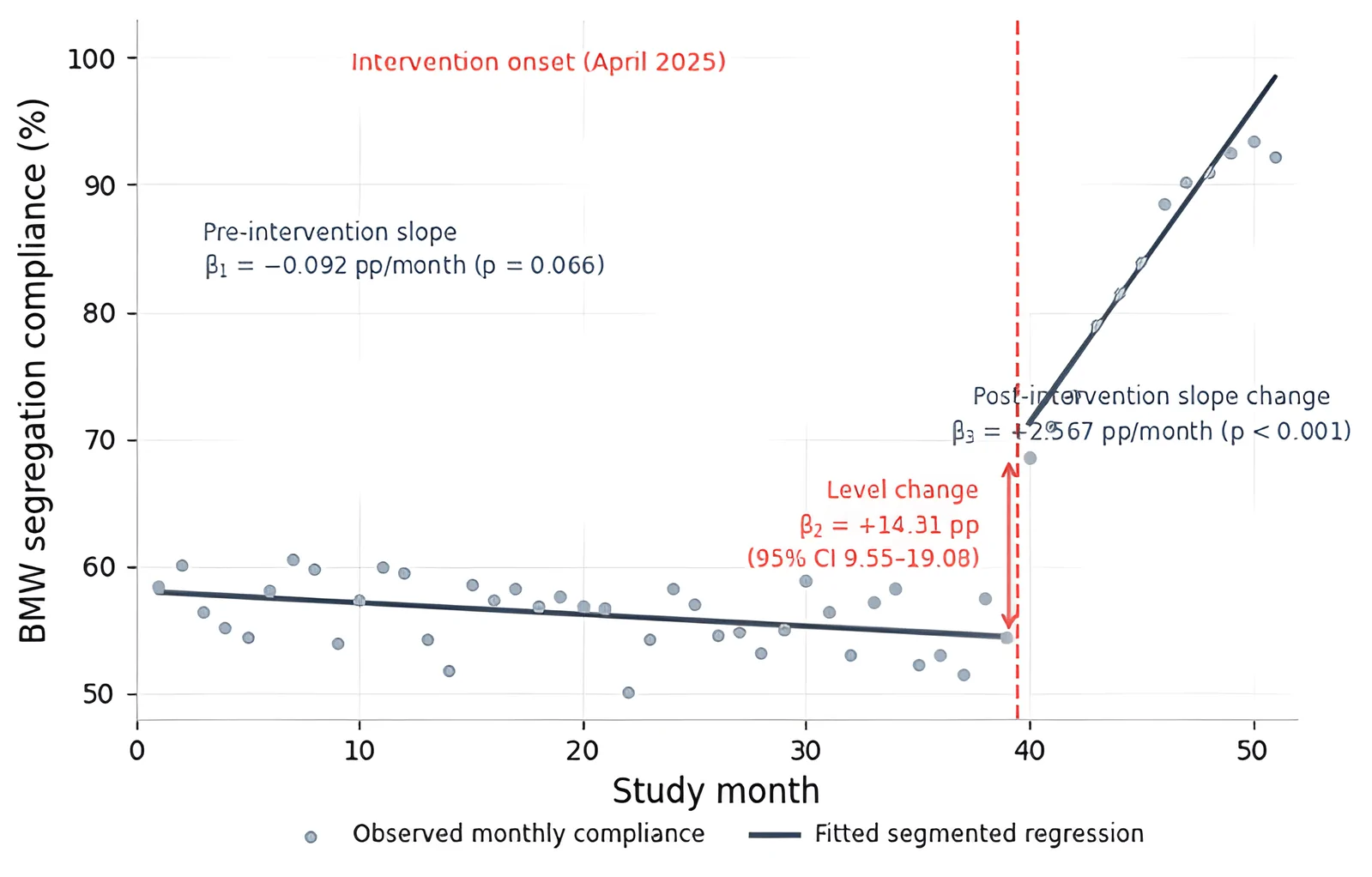
Interrupted time series of monthly BMW segregation compliance (months 1-51), with fitted segmented regression The intervention began at month 40 (April 2025). BMW: biomedical waste

Sensitivity analysis results

All four pre-specified sensitivity analyses yielded results consistent with the primary model.

Seasonal Harmonic Adjustment

Adding sin(2πt/12) and cos(2πt/12) terms produced β₂=14.09 (95% CI: 9.14-19.04; p<0.001) and β₃=2.543 (p<0.001), virtually unchanged from the primary model, indicating that the pre-intervention seasonal pattern does not drive the observed compliance improvement.

Lag Sensitivity

Shifting the assumed intervention onset one month earlier (March 2025) yielded β₂=13.92 (p<0.001) and one month later (May 2025) yielded β₂=14.54 (p<0.001); the slope change β₃ was similarly unchanged. The results are robust to plausible uncertainty in intervention timing.

Alternative Autocorrelation Specification

Prais-Winsten AR (1) correction and Newey-West lag 6 both produced β₂ estimates within 0.25 pp of the primary model, with overlapping confidence intervals. All specifications agreed that the level and slope changes were highly statistically significant.

NSI Model Sensitivity

Poisson, negative binomial, and zero-inflated Poisson models all produced non-significant β₂ and β₃ estimates for NSI (all p>0.27), consistent with the primary log-linear result. The non-significance of the NSI outcome is therefore not a modelling artifact.

NSI incidence

Monthly NSI counts showed a descriptive downward pattern after intervention onset (Table 5). During the 39-month pre-intervention period, 81 NSI events were recorded (mean: 2.08 per month; SD: 1.44), corresponding to an estimated rate of 1.13 events per 100 occupied bed-years (denominator: 2,210 occupied beds). This rate is substantially lower than the 8.9 and 10.4 events per 100 beds per year reported from Indian tertiary care surveillance studies [11,12], an expected finding given the well-documented under-reporting associated with passive manual incident registers [13]. During the six-month sustained phase, two NSI events were recorded (mean: 0.33 per month; SD: 0.52), corresponding to 0.18 events per 100 occupied bed-years.

**Table 5:** NSI incidence by study phase Rate=(total events×12)/(months×22.10), where 22.10=2,210 occupied beds/100. †The Poisson IRR compares the six-month sustained phase (two events) with the six months immediately preceding intervention, October 2024-March 2025 (10 events; 0.90 per 100 occupied bed-years), not with the full pre-intervention period; the unadjusted count ratio (2/10) is 0.20, and the model-based estimate with robust standard errors is 0.21. The IRR is vulnerable to small-n effects and regression to the mean. ITS log-linear model parameters for NSI: β₂ p=0.397 and β₃ p=0.270 (not statistically significant). The NSI finding must be treated as an associative trend only. No digital reporting system was operational; all data were derived from a manual paper-based incident register. NSI: needle-stick injury; PDSA: Plan-Do-Study-Act; ITS: interrupted time series; IRR: incidence rate ratio

Phase	Months	Total NSI	Mean/month	SD	Rate/100 occupied bed-years	Notes
Pre-intervention	39	81	2.08	1.44	1.13	Passive manual register; under-reporting expected
PDSA Cycle 1 (April-June 2025)	3	4	1.33	1.53	0.72	Training and infrastructure phase
PDSA Cycle 2 (July-September 2025)	3	4	1.33	2.31	0.72	Reinforcement; corrective actions
Sustained phase (October 2025-March 2026)	6	2	0.33	0.52	0.18	Poisson IRR=0.21 (95% CI: 0.06-0.78; p=0.020) vs. pre-period†

Importantly, the log-linear ITS model for NSI counts did not reach statistical significance: the level change at intervention onset was β₂=-0.320 (SE: 0.374; p=0.397), and the post-intervention slope change was β₃=-0.051 (SE: 0.046; p=0.270). Model fit was modest (R²=0.201), reflecting the variability inherent in low-frequency count data with multiple zero-count months. We therefore interpret the observed NSI reduction as an associative trend consistent with improved sharps segregation rather than as a confirmed intervention effect. The sustained phase yielded a Poisson IRR of 0.21 (95% CI: 0.06-0.78; p=0.020) relative to the six months immediately preceding intervention onset (October 2024-March 2025), during which 10 NSI events were recorded (mean: 1.67 per month; 0.90 per 100 occupied bed-years), compared with two events (0.33 per month; 0.18 per 100 occupied bed-years) in the sustained phase; the unadjusted count ratio is 0.20, and the model-based estimate with robust standard errors is 0.21. This provides descriptive support for a reduction but is subject to regression to the mean, small-number instability, and the confounding influence of a passive reporting system. Event counts and rates by study phase are shown in Figure 3.

**Figure 3:**
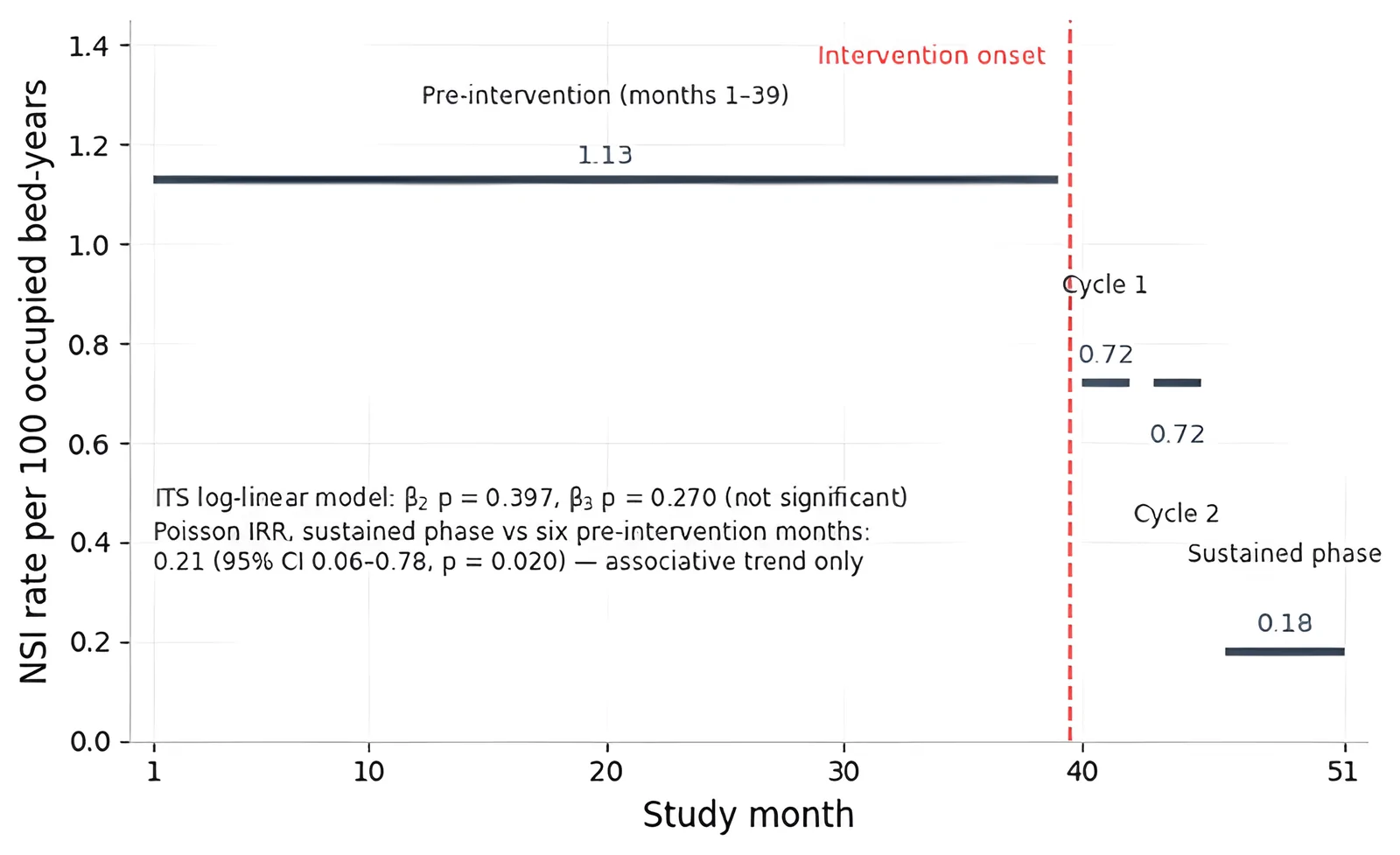
NSI rate per 100 occupied bed-years by study phase The decline was not statistically significant in the interrupted time series model and should be interpreted as an associative trend only. NSI: needle-stick injury; IRR: incidence rate ratio

Cadre-specific knowledge assessment

KAP scores improved significantly across all four cadres (n=312) following the intervention (Table 6). Medical doctors improved from 52.8% to 89.3% (+36.5 pp; Cohen's d=2.14; p<0.001), addressing a documented gap in physician engagement with waste management protocols. Nurses showed the smallest absolute gain (+20.5 pp; d=1.38; p<0.001) alongside the highest post-intervention score, consistent with a ceiling effect. Sanitation staff improved from 55% to 92.8% (+37.8 pp; d=2.31; p<0.001). Waste handlers, who had the lowest baseline scores and the highest occupational exposure, improved from 45.5% to 81.8% (+36.3 pp; d=1.97; p<0.001). The concentration of the largest gains in the cadres with the lowest baseline scores is consistent with the PDSA principle of addressing the point of greatest need. Cadre-specific score changes are shown in Figure 4.

**Table 6:** Cadre-specific knowledge assessment scores before and after the intervention (n=312) Pre-score: baseline before PDSA Cycle 1 training; post-score: immediately following PDSA Cycle 2 completion. Validated BMW knowledge questionnaire (5-domain, 100-point scale). Overall values are the cadre size-weighted pooled means. All comparisons two-tailed; α=0.05. BMW: biomedical waste; PDSA: Plan-Do-Study-Act; SR: signed rank; d: Cohen's d effect size; pp: percentage points

Cadre	n	Pre-score (%)	Post-score (%)	Δ (pp)	Cohen's d	Test	P-value
Medical doctors	68	52.8	89.3	+36.5	2.14	Paired t-test	<0.001
Nursing staff	96	78	98.5	+20.5	1.38	Wilcoxon SR	<0.001
Sanitation staff	84	55	92.8	+37.8	2.31	Wilcoxon SR	<0.001
Waste handlers	64	45.5	81.8	+36.3	1.97	Wilcoxon SR	<0.001
All cadres (overall)	312	59.7	91.5	+31.9	1.85	Paired t-test	<0.001

**Figure 4:**
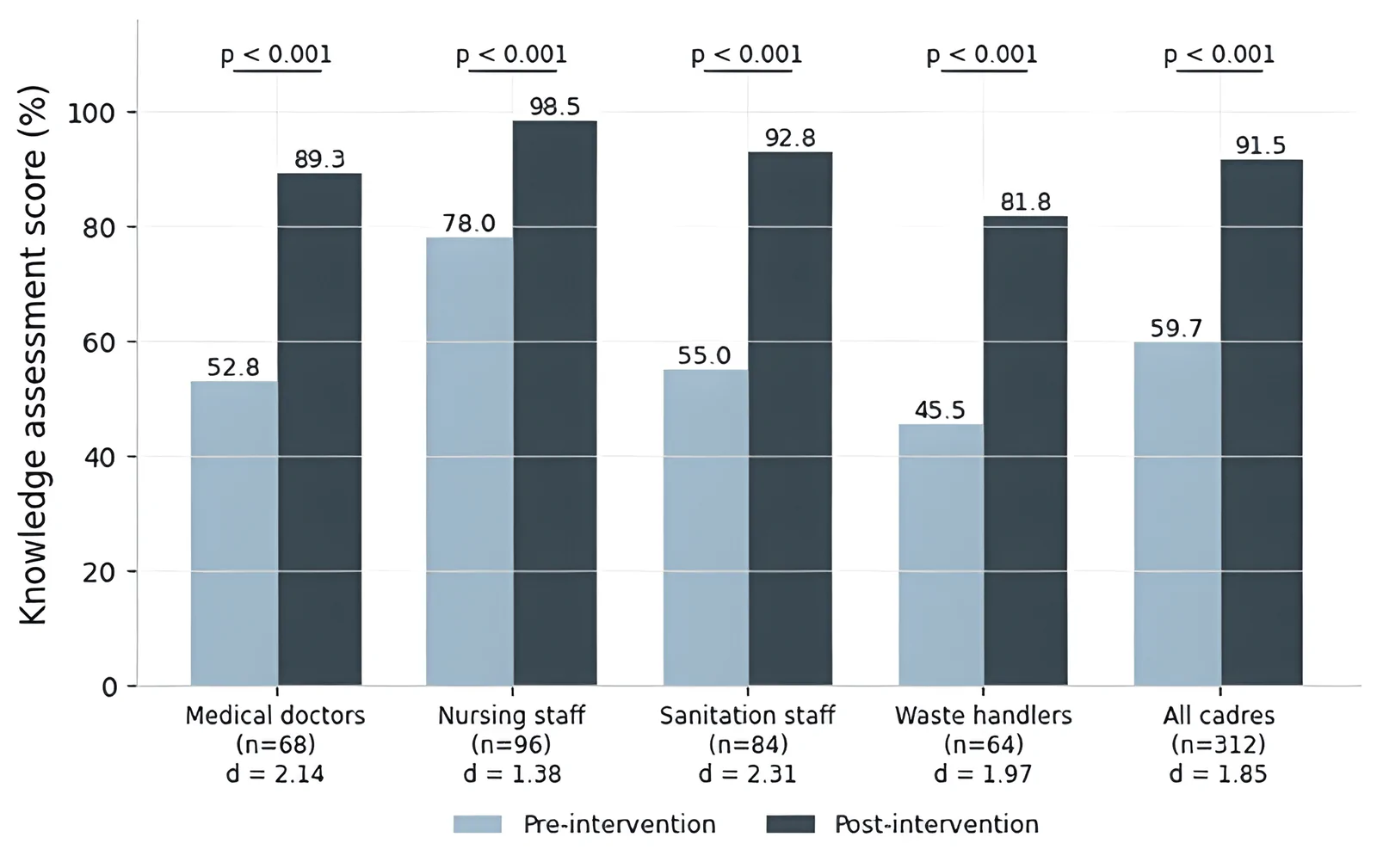
Cadre-specific knowledge assessment scores before and after the PDSA intervention (n=312) All comparisons: p<0.001; values are as reported in Table 6. PDSA: Plan-Do-Study-Act

Comparison with published Indian studies

Table 7 compares our results with published Indian tertiary hospital BMW compliance studies. The magnitude of our compliance trajectory (from approximately 56% at baseline to approximately 91% in the sustained phase) is comparable to the largest published Indian pre-post study, which reported an improvement from 57% to 91% in a tertiary care teaching hospital in Eastern India [6]. The analytical contribution of the present study lies in the 39-month pre-intervention baseline, which demonstrates institutional drift (-0.092 pp/month), and in the post-intervention slope, which shows continued acceleration (+2.57 pp/month), both estimated against that counterfactual trajectory. The nationwide gap assessment reported by Joseph et al. [5] documents persistent shortfalls in structured BMW implementation practices across Indian healthcare facilities.

**Table 7:** Comparison with published Indian BMW compliance studies *KAP score units (e.g., out of 10), not percentage compliance. †Percentage of the total achievable knowledge score (mean 14.00, SD 3.040, rising to 19.94, SD 1.990, out of a maximum of 25), as reported by the authors. ‡Reported in the source as ''p<0.000" and rendered here as p<0.001, because a probability cannot be less than zero. Joseph et al. [5] is a cross-sectional national survey without a pre-post comparison; selected facility-level practices are summarized instead. For the present study, "pre" means the mean of the 39-month pre-intervention period, and "post" means the mean of the six-month sustained phase; ITS estimates are from the primary segmented regression model. KAP: knowledge, attitude, and practice; BMW: biomedical waste; pp: percentage points; ITS: interrupted time series; PDSA: Plan-Do-Study-Act; NSI: needle-stick injury; NS: not significant; HCF: healthcare facility

Study	Setting	Pre (%)	Post (%)	Δ (pp)	Test/p-value	Design and key note
Sahoo et al., 2024 [6]	Tertiary care teaching hospital, Eastern India	57.0	91.0	+34.0	Paired t/p<0.00001	Pre-post; largest comparable gain
Singh et al., 2020 [10]	Tertiary care teaching institute, Rohtak, Haryana	56.0†	80.0†	+24.0	Paired t/p<0.001‡	Pre-post; n=250 healthcare professionals
Shivashankarappa et al., 2024 [8]	700-bed private hospital, Bengaluru	4.7*	8.8*	+4.1*	Wilcoxon/p<0.001	*KAP score units; pre-post
Pindi et al., 2023 [7]	Government tertiary hospital, Hyderabad	96.5	97.5	+1.0	p=0.4 (NS)	Ceiling effect at baseline
Joseph et al., 2025 [5]	Mixed HCFs (n=267), nationwide	-	-	-	Cross-sectional survey	67% accredited; 51% discarded needles directly into sharps containers, 43% used dedicated BMW tags, 58% fully implemented barcoding
Present study (2026)	Government tertiary teaching hospital, Jharkhand (2,327 beds, 95% occupancy)	56.2	91.3	+35.1 descriptive (+14.3 pp ITS level change)	ITS β₂ p<0.001; β₃ p<0.001	51-month ITS; PDSA; NSI co-primary; sensitivity analyses conducted

Consolidated statistical summary

A consolidated summary of the primary, co-primary, and main secondary outcome analyses is presented in Table 8 (cadre-specific results appear in Table 6), and the corresponding effect estimates are displayed in Figure 5.

**Table 8:** Consolidated statistical summary of all outcomes *Mean of the 39-month pre-intervention period. **Mean of the six-month sustained phase. †Sustained phase (October 2025-March 2026). ‡The NSI Poisson IRR comparison window is the six months immediately preceding intervention (October 2024-March 2025; 10 events; 0.90 per 100 occupied bed-years), not the full 39-month pre-intervention period; the unadjusted count ratio (2 vs. 10 events) is 0.20, and the model-based robust standard error estimate is 0.21. Cadre-specific knowledge results are presented in Table 6. Caution should be exercised when interpreting the NSI Poisson IRR: small denominator (two events in the sustained phase), passive reporting system with no digital support, and risk of regression to the mean. BMW: biomedical waste; ITS: interrupted time series; pp: percentage points; NSI: needle-stick injury; KAP: knowledge, attitude, and practice; IRR: incidence rate ratio; occ-yr: occupied bed-years

Variable	Test/model	Pre	Post	Effect size	Statistic	P-value
BMW compliance (%)	ITS: level change (β₂) (primary)	56.2 (2.6)*	91.3 (1.9)**	+14.3 pp	t=6.04	<0.001
BMW compliance (%)	ITS: slope change (β₃) (primary)	-	-	+2.57 pp/month	t=8.82	<0.001
NSI monthly count	ITS log-linear: level (β₂) (not significant)	2.08 (1.44)	0.33 (0.52)†	β₂=-0.320	z=-0.86	0.397
Overall KAP (%)	Paired t-test (secondary)	59.7	91.5	+31.9 pp; d=1.85	-	<0.001
NSI (6-month sustained phase vs. 6 months immediately preceding intervention)	Poisson IRR (associative only, not primary)	0.90/100 occ-yr‡	0.18/100 occ-yr	IRR=0.21 (0.06-0.78)	χ²=5.41	0.020

**Figure 5:**
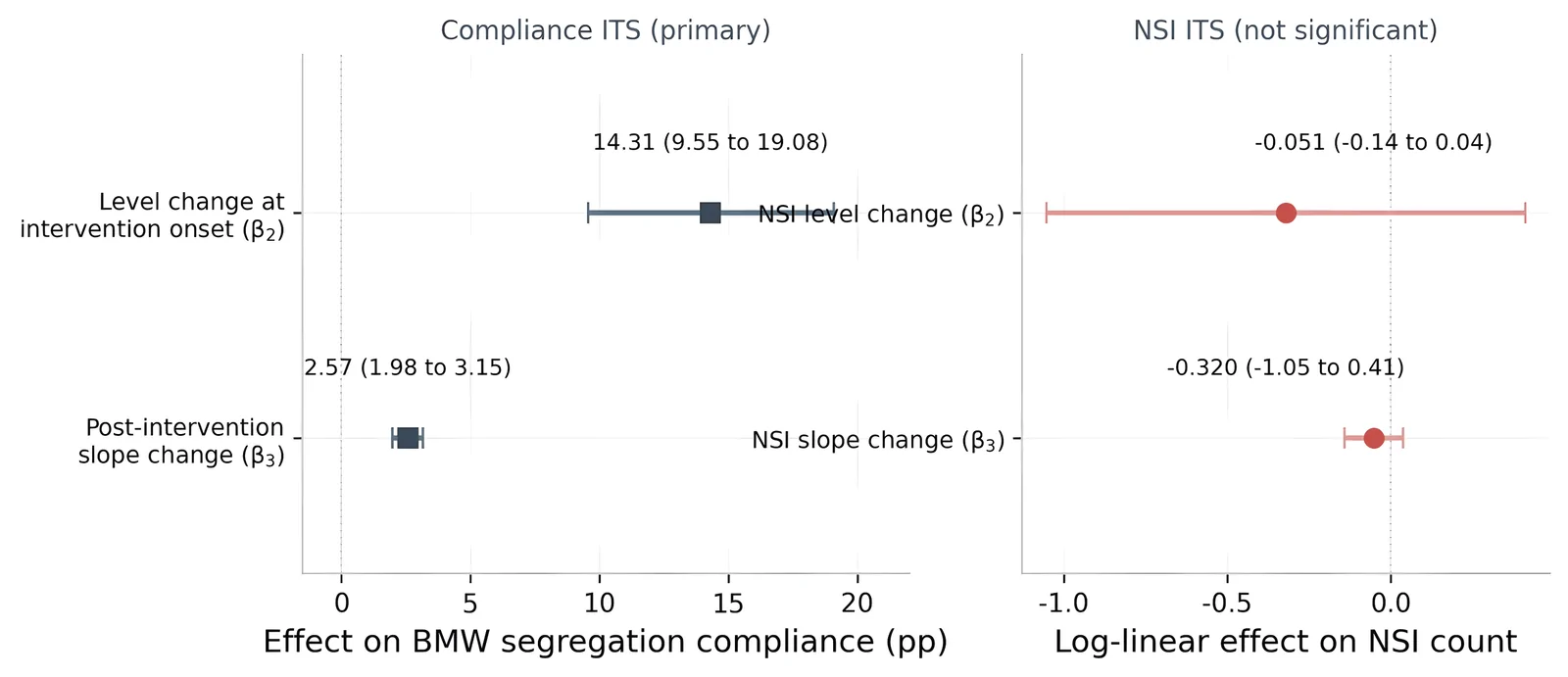
Forest plot of effect estimates with 95% CI for the primary compliance outcomes and the non-significant NSI outcomes BMW: biomedical waste; NSI: needle-stick injury; ITS: interrupted time series

## Discussion

Main findings

This 51-month ITS study found a large, statistically significant, and sustained increase in BMW segregation compliance following a two-cycle PDSA intervention in a 2,327-bed government tertiary teaching hospital. The improvement was robust across all four sensitivity analyses and occurred against a pre-intervention baseline of institutional drift, indicating a genuine change in trajectory rather than the continuation of an existing trend. By the end of the sustained phase, compliance was approximately 91%.

NSI incidence declined descriptively over the same period, but the decline was not statistically significant in the ITS model, an expected result given the low event frequencies, zero inflation, and passive manual reporting. We therefore present the NSI finding as an associative, hypothesis-generating trend for future studies with active surveillance.

Comparison with published literature

The compliance trajectory observed here, from approximately 56% to approximately 91%, sits at the upper bound of published Indian intervention studies. Sahoo et al. reported a comparable 34-pp improvement in a tertiary care teaching hospital in Eastern India using a pre-post design [6], and Singh et al. reported a 24-pp gain in mean knowledge score (from 56% to 80% of the total achievable score) among healthcare professionals at a North Indian tertiary teaching institute following structured training [10]. Both studies used short pre-post intervals that cannot establish pre-existing trends. The Hyderabad study by Pindi et al. (from 96.5% to 97.5%, non-significant) illustrates the ceiling effect encountered in high-baseline institutions, underscoring that the magnitude of improvement depends heavily on baseline performance [7]. The nationwide gap assessment by Joseph et al. confirms that structured BMW practices remain inconsistently implemented across Indian healthcare facilities [5]. The principal analytical advantage of the present study over these benchmarks is the 39-month baseline, which establishes a near-flat counterfactual and strengthens the temporal inference linking the post-April 2025 improvement to the PDSA intervention.

Possible mechanisms

The observed compliance improvement is consistent with several plausible mechanisms. First, the structured two-cycle design addressed both knowledge (training) and environment (infrastructure, bin replacement, signage), the two most commonly reported failure modes in waste management programs [16,17]. Second, the BMW Quality Committee, with fortnightly meetings and department-level scorecard feedback, institutionalized accountability in a self-reinforcing cycle. Third, the knowledge gains among the highest-exposure cadres with the lowest baseline knowledge (d≥1.97) are consistent with the PDSA principle of addressing the point of greatest need. Fourth, the positive post-intervention slope suggests that the PDSA architecture produced not only a one-time structural change but a continuing trajectory of improvement, which a purely structural change would not be expected to generate.

Strengths of the analytical approach

The 51-month ITS design offers several advantages over short pre-post comparisons. First, ITS uses the hospital's own pre-intervention trend as an internal counterfactual, reducing dependence on external comparison groups [20,21]. Second, the 39-month pre-intervention baseline provides reasonable statistical precision for the pre-intervention trend, which shorter studies cannot achieve. Third, the 12 post-intervention months allow the assessment of whether effects decay, persist, or accelerate information directly relevant to policymakers deciding how long such interventions should be maintained. Fourth, the compliance finding is supported by four pre-specified sensitivity analyses and therefore does not rest on a single modelling decision [22]. Fifth, the explicit separation between the statistically confirmed compliance result and the merely associative NSI result is methodologically sound reporting.

Limitations and sources of bias

The limitations of this study are presented as the main methodological context of all findings, not as something at the end. The main limitation for the causal inference of ITS will be the absence of a concurrent control group.

During 2024-2025, the Jharkhand State Health Project Coordination Unit (HPCU) undertook an intensive drive to make the different government medical colleges facility-wise NABH accreditation ready. They did unannounced sanitation inspections and sanitation contract renewal as part of this. These measures could have raised the expectations and performance of compliance, respectively. While the ITS model shows a step change in April 2025, the pre-intervention period was not unregulated, restricting the ability to disentangle the effect of the PDSA intervention from concurrent state-level accreditation pressure.

Seasonal and Staffing Variation

Patient census peaks during the monsoon months (July-September) and in December. The 12-monthly junior medical officer rotation cannot plausibly confound the intervention effect: the cohort in post at intervention onset had arrived in April 2024, 12 months before the intervention began in April 2025. Staffing ratios are not explicitly included as monthly covariates in the ITS model, and nursing vacancy rates ranged from 18% to 34% over the study period. The seasonal sensitivity analyses did not materially alter the main estimates.

NSI Under-Reporting and Reporting System Limitations

Sharps injuries are well known to be under-reported, with estimates suggesting that half or more of events go undocumented [13]. All NSI data in this study were derived from a manual, paper-based register; no digital incident reporting system was operational at any point. If reporting completeness improved over the study period, for example, because PDSA training raised awareness of the importance of reporting, any true decline in NSI events would be partially masked by more complete ascertainment, biasing the observed reduction toward the null. Conversely, heightened early awareness may have improved documentation before compliance gains reduced event counts. These opposing effects cannot be separated without a digitized reporting system.

Audit Scoring and Observer Bias

Although conducted by trained assessors with documented inter‐rater agreement, compliance audits may be affected by unconscious scoring drift after auditors had a role in intervention design. Keeping the same audit team throughout minimizes measurement drift but does not eliminate observer bias. The data is presented in the form of an objective weight measurement and is free of this source of error. It is also important to note that the assessment of inter-rater reliability was not independent as the three auditors were trained as a team and therefore may be biased. Another limitation associated with this is that the weighbridge logs only record total BMW weight and annual stream-wise waste weights were not weighed separately for each calendar year; Table 3 stream-level figures are estimates apportioned from the measured annual totals using year-specific audit-derived stream shares and should not be read as independent yearly measurements.

Interpretation of the Coefficient of Determination

The segmented ITS time for structure, intervention, and phase, with R²=0.939, explains almost all the systematic variation in the compliance series. This does not mean it is unlikely that there are unmeasured confounders, nor does it prove causality. A high R² for a short time series model is likely the result of model parameterization rather than real causal isolation and therefore should be interpreted strictly as evidence of model fit and not evidence against confounding.

Generalizability

It is a single-institution study with a unique administrative and cultural profile. The results from a government tertiary hospital with a high occupancy of 95% and high pressure situated in Jharkhand, serving mostly a tribal population and facing chronic public sector staffing shortages, may not be generalizable to metropolitan tertiary centers, smaller facilities, and organizations with a higher baseline compliance.

Policy implications

Although there are many limiting factors, the strength and durability of compliance improvement are of practical importance. Institutional compliance improved significantly from nearly 56% to about 91%. This 35-pp gain brought the hospital closer to NABH accreditation readiness [4]. During the study, it was institutionalized to operate a fortnightly BMW Quality Committee, use departmental scorecards, and conduct cadre-stratified retraining. This is easily transferable to other government hospitals [16,17]. The current results suggest that the group of workers that should be prioritized for training investment in under-resourced public hospitals is waste handlers and sanitation staff, who showed the highest gain in knowledge.

These findings support the consideration of the PDSA-based BMW monitoring framework in resource-constrained public hospitals and may inform the design of future multi-center implementation. The future NABH quality standards may give preference to planned and monitored QI mechanisms for BMW management instead of a one-time training and infrastructure audits [4]. A formal NSI incidence rate per 100 occupied bed-years, which would be tracked alongside BMW compliance, could, with other data, become a linked quality indicator. It would require investigation, from passive manual reporting to active digital surveillance. The latter was not available in the current setting [13].

Future research directions

A valuable methodological advance for this field would be a multi-center stepped-wedge cluster randomized trial in which hospital units or campuses receive the intervention in randomized order [21]. Such a design would preserve the implementation-science logic of the PDSA framework while providing a concurrent controlled comparison at each time point. Alternatively, synthetic control methods could construct a credible counterfactual for treated hospitals from multiple non-intervened hospitals with comparable baseline trajectories [22]. For the NSI outcome, future studies will require active surveillance through validated digital reporting systems that capture near-misses and unreported events, allowing a direct test of whether improved segregation causally reduces sharps exposure [11,12]. Finally, post-intervention follow-up of at least 24 months is needed to determine whether compliance gains persist beyond the period of active Quality Committee monitoring.

## Conclusions

A two-cycle PDSA QI intervention was associated with a large, statistically significant, and sustained increase in BMW segregation compliance in a large government tertiary teaching hospital in Eastern India over 51 months of prospective observation, robust across four sensitivity analyses and observed against a pre-intervention baseline of institutional drift. The descriptive decline in NSIs was not statistically significant in the time series model and should be interpreted with caution, given the passive reporting through a manual incident register. Knowledge gains were significant across all staff cadres and were largest among frontline waste handlers and sanitation workers.

Because no concurrent control group was available, the compliance improvement cannot be attributed to the intervention alone; state-level accreditation pressure, staff turnover, and possible audit scoring drift may all have contributed. Within these limits, the study offers a transferable PDSA-based structured facility audit protocol, bilingual information and education materials, cadre-stratified training, infrastructure reinforcement, and an active Quality Committee with departmental scorecards for resource-constrained government hospitals. Validation in multi-center-controlled designs and the incorporation of active digital NSI surveillance are the priorities for strengthening the evidence base.
